# Comparison of the effects of three different resistance training methods on muscle fatigue in healthy untrained men

**DOI:** 10.3389/fspor.2024.1497979

**Published:** 2024-11-21

**Authors:** Masafumi Kadota, Masatoshi Nakamura, Riku Yoshida, Kosuke Takeuchi

**Affiliations:** ^1^ Department of Physical Therapy, Kobe International University, Kobe-shi, Japan; ^2^ Faculty of Rehabilitation Sciences, Nishi Kyushu University, Kanzaki-cho, Japan; ^3^ Department of Rehabilitation Medicine, Maniwa Orthopedics Clinic, Niigata, Japan

**Keywords:** resistance training, fatigue, rest interval, order, training volume

## Abstract

**Introduction:**

Traditional set (TS), paired-set (PS), and super-set (SS) are used as resistance training methods. However, the effects of these methods on muscle fatigue (muscle strength and training volume) are not clear. The purpose of this study was to compare the effects of TS, PS, and SS on the muscle fatigue of the hamstrings and quadriceps.

**Methods:**

Thirteen healthy, untrained men performed three sets of leg curl and leg extension exercises. TS included three successive sets of the leg curl and leg extension exercises with a 60 s rest interval between sets and exercises. In the PS, leg curl exercises were performed alternatingly with the leg extension exercises with a 60 s rest interval between sets and exercises. In the SS, leg curl and leg extension exercises were performed alternatively with each set. During SS, a 60 s rest interval was set between sets but not between exercises. Muscle strength before and immediately after interventions, and training volume during the training, were measured using an isokinetic dynamometer machine. Time efficiency was calculated by dividing the total training volume by the time required for each intervention.

**Results and discussion:**

The muscle strength of the hamstrings decreased in PS (*p* = 0.039) and SS (*p* = 0.001) but did not change in TS (*p* = 0.434). Muscle strength of the quadriceps decreased in all interventions (*p* < 0.05). In all interventions, the training volume of the hamstrings decreased in Set 2 (*p* < 0.05), and that of the quadriceps decreased in Set 3 (*p* < 0.05). The total training volume in PS was higher than TS (*p* < 0.01) and SS (*p* = 0.03). Time efficiency in SS was higher than TS (*p* < 0.01) and PS (*p* < 0.01). These results indicated that PS could be useful for individuals with sufficient time for resistance training because of greater training volume, while SS could be useful for those with limited time due to better time efficiency.

## 1 Introduction

Resistance training is often used to improve athletic performance in athletes and health conditions in the general population (1–4). It is useful for muscle hypertrophy, muscle strengthening, and improving maximal power and muscle endurance (1, 3, 4). It is also used to reduce pain, improve functional capacity in patients with musculoskeletal disorders, and improve symptoms and psychosomatic function, such as increasing bone density in the elderly (5, 6).

Resistance training is characterized by load intensity, number of sets, number of repetitions, rest intervals, exercise order, load displacement, and time under tension, etc. (7–10). Even if resistance training is performed with a similar number of sets and repetitions, its order and rest intervals influence its effectiveness (11, 12). There are two procedures regarding the order of resistance training: (1) successive training (i.e., completing all training sets for a given exercise before performing the next training exercise) or (2) alternative training (i.e., performing agonist and antagonist muscle resistance training alternating with each set). A traditional set (TS) is widely used as a successive resistance training method (13), but paired-set (PS) and super-set (SS) are used as alternative methods (13, 14). SS trains agonist and antagonist muscles alternatively with limited or no inter-set rest interval (8, 13). Theoretically, in single-joint movements, when antagonist muscles are being trained, the antagonist muscles are not contracted and are in a rest-like state (11, 15, 16). TS trains one muscle continuously, while PS trains the agonist and the antagonist muscles alternately. Therefore, even if the same conditions (number of repetitions, number of sets, and rest interval) are set for TS and PS, the actual rest interval of the muscle in PS is longer than in TS.

A rest interval is an essential variable of resistance training because it directly influences muscle and general fatigue, muscle recovery, and training duration (12, 17, 18). The rest interval influences the metabolic response during resistance training, and the rest interval duration should be sufficient to remove the accumulation of lactic acid (18). If the rest interval is too short, it increases dependence on glycolytic energy production and may affect metabolic accumulation (18). Therefore, sufficient rest intervals are essential for sustaining repeated high-force muscle contractions and increasing muscle strength (5). However, because TS, PS, and SS have different rest intervals under the same conditions (repetitions and sets), it is necessary to clarify the effect of each resistance training protocol on muscle fatigue.

Muscle fatigue is assessed using muscle strength measurements before and after resistance training, and changes in training volume during resistance training (19, 20). Robbins et al. (20) compared the effects of SS and TS in trained men and found no difference in the change in the training volume (repetitions loads) during resistance training between SS and TS. On the other hand, Paz et al. (7) compared SS and TS in recreationally trained men and reported a more significant reduction in training volume during resistance training in TS than in SS. Additionally, Paz et al. (9) reported that total training volume (repetitions × sets × loads × exercises) was higher for PS and SS than TS in trained men. However, they did not assess muscle fatigue (9). Therefore, TS, SS, and PS have different effects on muscle fatigue, but no consensus has been reached. The difference between these widely used training protocols in muscle fatigue should be examined in detail to develop an effective resistance training method.

Therefore, the purpose of this study was to compare the effects of TS, PS, and SS on muscle fatigue in untrained, healthy young men. This study hypothesizes that muscle fatigue is lesser in PS than in TS and SS because PS has the most extended actual rest interval. In addition, SS has the highest time efficiency (training volume per minute) based on a previous study (20).

## 2 Materials and methods

### 2.1 Experimental approach to the problem

**Figure 1:**
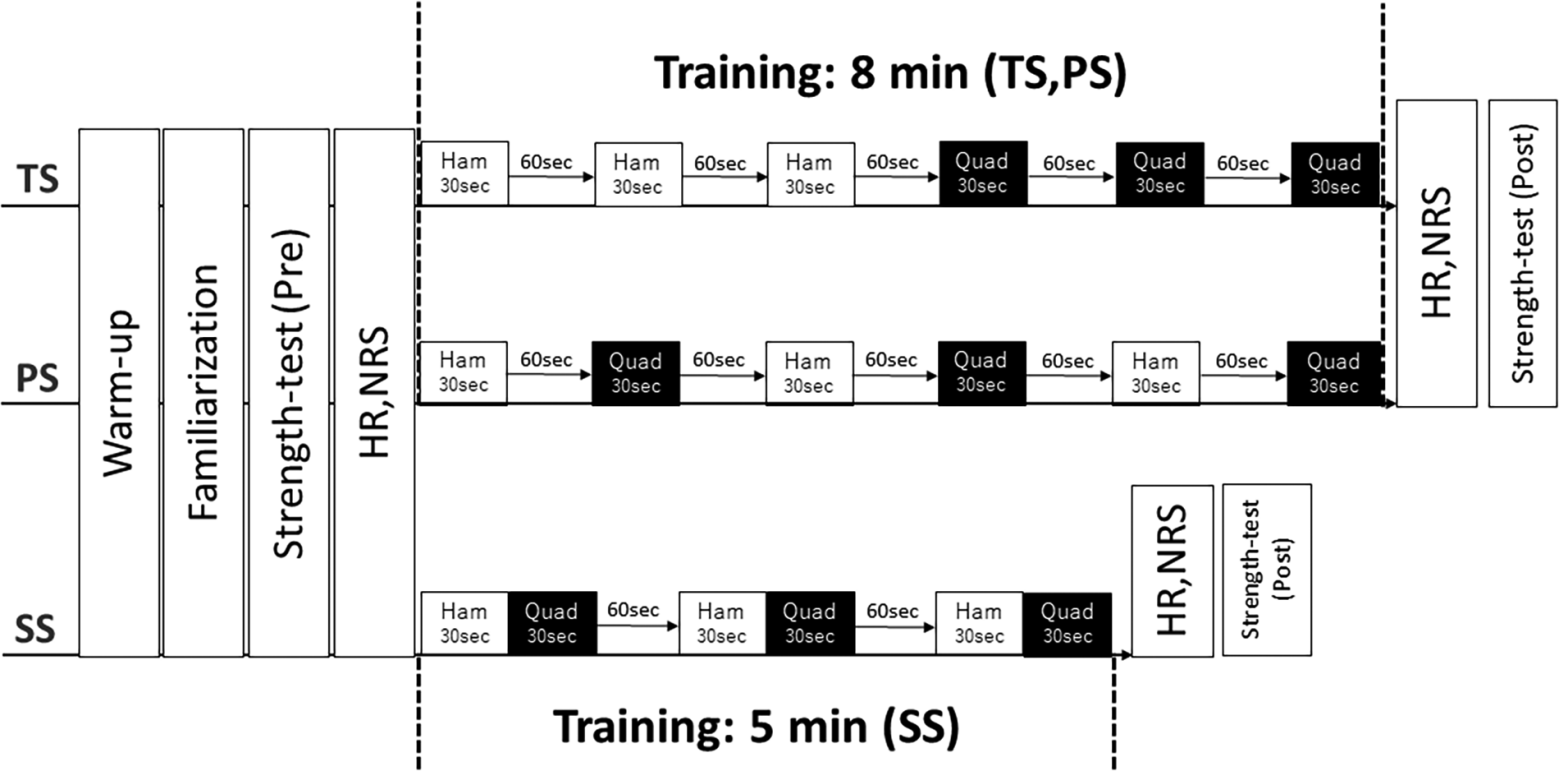
Experimental protocol. TS, traditional set; SS, superset; PS, paired-set; HR, heart rate; NRS, numerical rating scale; Ham, hamstrings; Quad, quadriceps.

In the present study, a randomized cross-over design was used. The participants underwent three different resistance training interventions with an interval of ≥2 weeks between visits. The experimental procedure was as follows: 5 min warm-up with a cycling ergometer (60 W, 120 bpm), familiarization session, pre-strength test, resistance training interventions, and post-strength measurement (Figure 1). In the familiarization session, the participants practiced isokinetic contraction exercises of the quadriceps and hamstrings with light loads. To assess muscle fatigue of the quadriceps and hamstrings in the dominant limb (ball kicking preference) (21), muscle strength before and immediately after training and training volume during the training were assessed. In addition, subjective muscle fatigue, subjective general fatigue, delayed onset muscle soreness (DOMS), and heart rate were assessed. The experiment was performed in a university laboratory, where the temperature was maintained at 24°C.

### 2.2 Participants

Thirteen healthy, active, but untrained male university students participated in this study (20.0 ± 0.4 years, 169.0 ± 5.3 cm, 62.3 ± 10.7 kg). Participants with a history of pathology in the quadriceps or hamstrings within 6 months were excluded. The sample size was calculated with a power of 80%, alpha error of 0.05, and effect size of 0.25 (middle) using G*Power 3.1 software (Heinrich Heine University, Düsseldorf, Germany), and the results showed that the requisite number of participants for this study was 12 participants; thus, 13 participants were recruited to account for possible attrition. All participants were informed of the requirements and risks associated with their involvement in this study and signed a written informed consent document. The study was performed in accordance with the Declaration of Helsinki (1964). The Ethics Committee of X approved the study (Procedure #G2023-186).

### 2.3 Muscle strength test

Muscle strength was measured using an isokinetic dynamometer machine (CYBEX NORM, Humac, California, USA). The participants sat on the dynamometer, and the angle between the backrest and the seat was set at 100 degrees. The trunk and thigh of the dominant leg were firmly secured with straps. The knee joint was aligned with the axis of rotation of the isokinetic dynamometer machine. The lever arm attachment was placed just proximal to the malleolus medialis and stabilized with straps. The range of motion of the knee joint during the muscle strength measurement was 0°–90° knee flexion, and the angular velocity was 60° /sec. The muscle strength of the hamstrings (leg curl) was measured first, followed immediately by the quadriceps (leg extension). The participants were instructed to perform maximum voluntary isokinetic concentric contractions. The greatest value of three maximum contractions was used for the analyses.

### 2.4 Resistance training interventions

The participants were secured on the isokinetic dynamometer machine in the same fashion as the muscle strength measurement. Resistance training interventions for the hamstrings (leg curl exercise) and quadriceps (leg extension exercise) were performed for 3 sets of 10 repetitions each. The range of motion of each training was 0°–90° knee flexion, and the angular velocity was 60° /sec. The participants were instructed to perform maximum voluntary isokinetic concentric and eccentric contractions. Participants rested in a seated position on the dynamometer machine during rest intervals. Because the previous study recommended short (<60 s) to moderate (i.e., 60–120 s) rest intervals for untrained participants (18), this study set a 60 s rest interval.

*TS.* Three sets of leg curl exercises were followed by 3 sets of leg extension exercises, with 60 s of rest interval between each exercise. The time required to perform the TS was approximately 8 min.

*PS*. Three sets of leg curl exercises were performed alternatingly with the 3 sets of leg extension exercises with 60 s of rest interval between each exercise. The time required to perform the PS was approximately 8 min.

*SS*. Leg curl exercises and leg extension exercises were performed alternatively with each set. There was no rest interval after the leg curl, but there was a 60 s rest interval after the leg extension. The time required to perform the SS was approximately 5 min.

### 2.5 Training volume

Knee flexion and extension torques during resistance training interventions were recorded with the dynamometer machine. Training volume was defined as the areas under the torque-time curve of each contraction, and it was calculated by integrating the torque (22). The training volume per set (10 repetitions) and total training volume (30 repetitions) were calculated for each quadriceps and hamstrings. Time efficiency was calculated by dividing the total training volume by the total time of resistance training interventions (8 min for TS and PS, 5 min for SS) (20).

### 2.6 Numerical rating scale

Subjective muscle fatigue, subjective general fatigue, and DOMS were quantified by an 11-point numerical rating scale (NRS) that ranged from 0 (no fatigue or no pain) to 10 (worst imaginable fatigue or pain) (23). Muscle and general fatigue were evaluated immediately after resistance training interventions, and DOMS was assessed 24 and 48 h later. DOMS was defined as pain with active knee flexion and extension.

### 2.7 Heart rate

To evaluate the changes in heart rate before and immediately after resistance training interventions, a Polar H10 sensor chest strap device (Polar Electro Oy, Kempele, Finland; sampling rate: 1,000 Hz; app software: Elite HRV App, Version 5.5.1) was used. Heart rate was recorded throughout the experiment, and data immediately before and after the resistance training interventions were used for analysis.

### 2.8 Statistical analyses

The Shapiro–Wilk test was used to assess normal distribution. NRS data for muscle fatigue and general fatigue were not normally distributed, but the other valuables were normally distributed. For the muscle strength, subjective muscle fatigue, subjective general fatigue, and heart rate data, a repeated two-way ANOVA (time [pre vs. post] × interventions [TS vs. PS vs. SS] was used. For the training volume data, a repeated two-way ANOVA [time [set 1 vs. set 2 vs. set 3] × interventions [TS vs. PS vs. SS]] was used. A two-way repeated measures ANOVA was used for DOMS data to examine the effects of interventions (TS vs. PS vs. SS) and time (24 h vs. 48 h). For the total training volume and time efficiency data, a one-way repeated ANOVA was used to analyze the difference between the interventions (TS vs. PS vs. SS). A one-way repeated ANOVA was used to analyze differences in baseline data between interventions (TS vs. PS vs. SS). If a significance was detected, *post hoc* analyses using Bonferroni's test were performed. Partial *η*^2^ values were reported to reflect the magnitude of the differences for each treatment (small = 0.01, medium = 0.06, and large = 0.14) (24). The statistical power was calculated from the effect size of the muscle strength and training volume, which were the main outcomes of this study, using G power software at a setting of *α* = 0.05 and a sample size of 13. The results indicated that the statistical power was 0.93–1.00. The analyses were performed using SPSS version 25 (SPSS, Inc., Chicago, IL, USA). Differences were considered statistically significant at an alpha of 0.05.

## 3 Results

### 3.1 Baseline data

There was no significant difference in variables between interventions (Table 1).

**Table 1:** Baseline data.

	TS	PS	SS	*p*-value
Muscle strength of the quadriceps at pre-value (Nm)	114.4 ± 29.1	131.0 ± 29.1	123.7 ± 32.0	0.184
Muscle strength of the hamstrings at pre-value (Nm)	66.3 ± 10.4	75.1 ± 12.3	71.9 ± 15.2	0.170
Training volume of the hamstrings in Set 1 (Nm)	1,213.5 ± 220.5	1,351.7 ± 239.8	1,269.8 ± 277.6	0.127
Subjective muscle fatigue at pre-value	2 (2–3)	3 (2–3)	2 (1–2)	0.128
Subjective general fatigue at pre-value	2 (1–3)	2 (1–2)	1 (0–2)	0.142
Heart rate at pre-value (bpm)	98.9 ± 12.9	96.7 ± 15.6	100.8 ± 15.1	0.697

Values except for subjective muscle fatigue, subjective general fatigue, and heart rate were described as mean ± standard deviation. Values for subjective muscle fatigue and subjective general fatigue were described as median (interquartile range). TS, traditional set; PS, paired-set; SS, super-set.

### 3.2 Muscle strength

**Figure 2:**
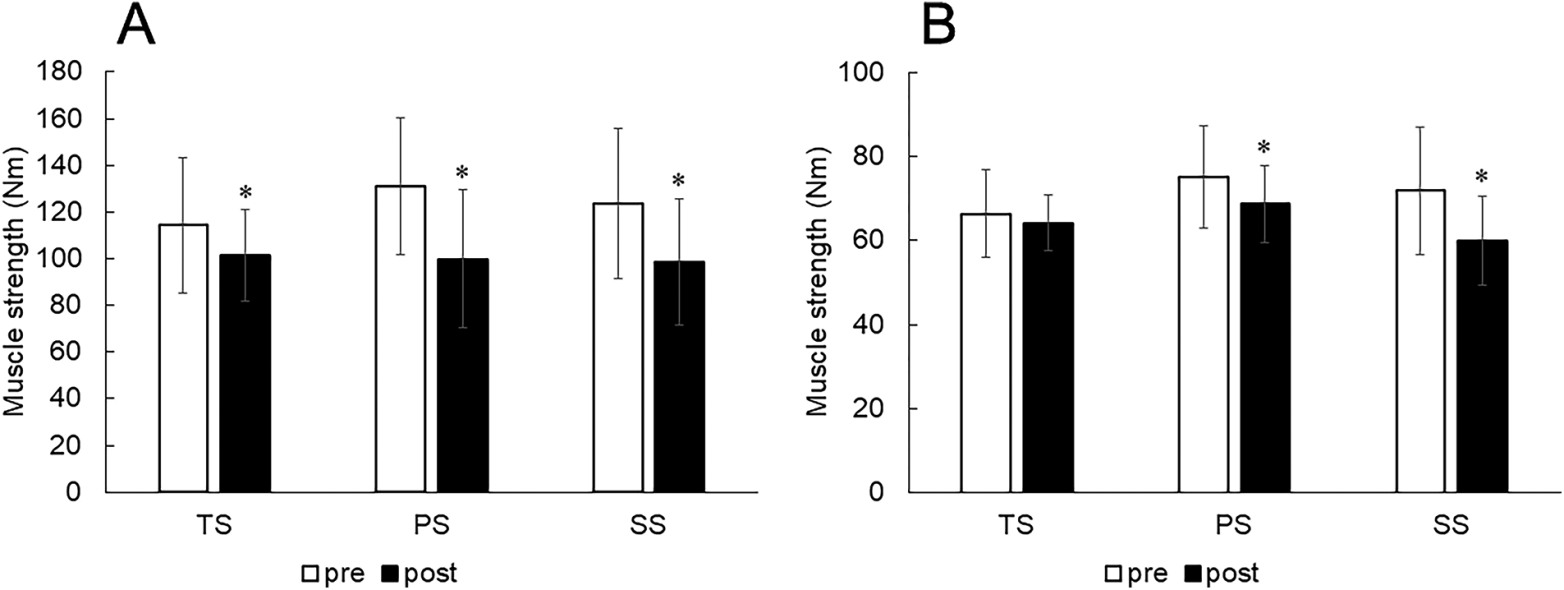
Change in muscle strength of the quadriceps **(A)** and hamstrings **(B)** values were described as mean ± standard deviation. *p < 0.05 vs. pre-value. PS, paired-set; SS, super set; TS, traditional set.

For the quadriceps, there was no significant interaction (*p* = 0.068, partial *η*^2^ = 0.201) and no main effect for interventions (*p* = 0.343, partial *η*^2^ = 0.085), but there was a significant main effect for time (*p* < 0.01, partial *η*^2^ = 0.502) (Figure 2). The muscle strength of the quadriceps significantly decreased after interventions (*p* < 0.01).

For the hamstrings, there was a significant interaction (*p* = 0.021, partial *η*^2^ = 0.274) (Figure 2). The muscle strength of the hamstrings decreased in PS (*p* = 0.039) and SS (*p* = 0.001) but did not change in TS (*p* = 0.434).

### 3.3 Training volume

**Figure 3:**
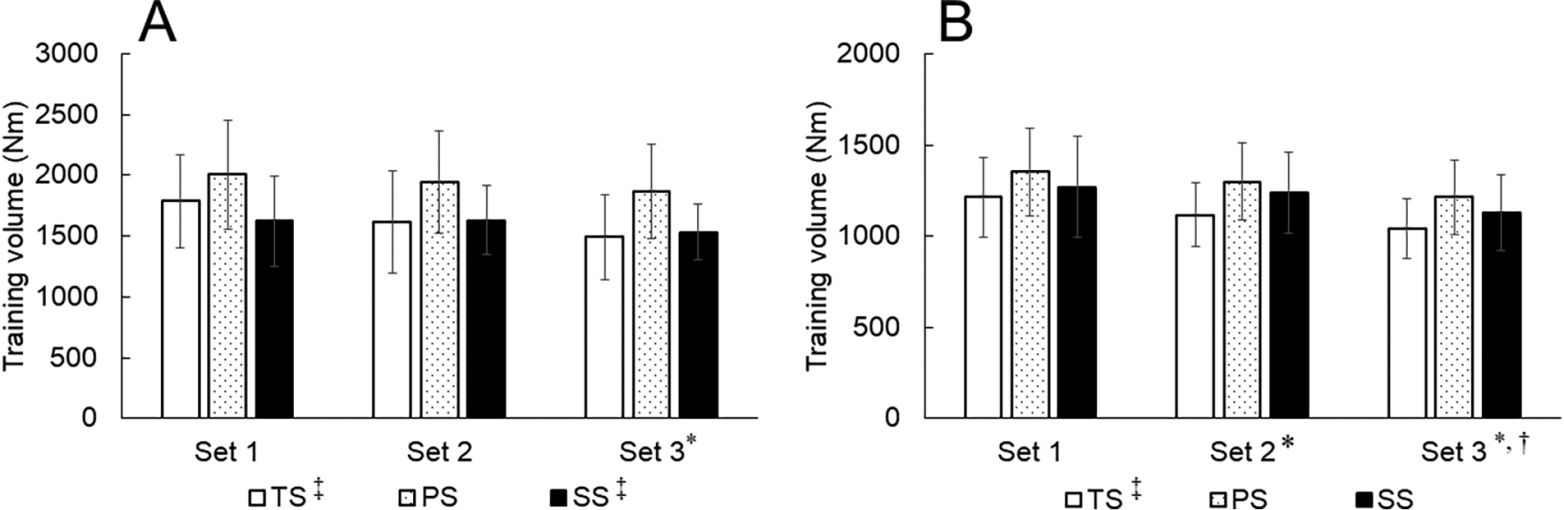
Change in the training volume of the quadriceps **(A)** and hamstrings **(B)** values were described as mean ± standard deviation. *p < 0.05 vs. Set 1. ^†^*p* < 0.05 vs. Set 2. ^‡^*p* < 0.05 vs PS. PS, paired-set; SS, super set; TS, traditional set.

For the quadriceps, there was no significant interaction (*p* = 0.092, partial *η*^2^ = 0.151), but there was a significant main effect for time (*p* < 0.01, partial *η*^2^ = 0.397) and interventions (*p* < 0.01, partial *η*^2^ = 0.393) (Figure 3). Set 1 was higher than Set 3 (*p* = 0.027). There was no significant difference between Set 1 and Set 2 (*p* = 0.091) or Set 2 and Set 3 (*p* = 0.104). PS was greater than SS (*p* = 0.033) and TS (*p* < 0.01). There was no significant difference between SS and TS (*p* > 0.99).

For the hamstrings, there was no significant interaction (*p* = 0.450, partial *η*^2^ = 0.072), but there was a significant main effect for time (*p* < 0.01, partial *η*^2^ = 0.723) and interventions (*p* = 0.011, partial *η*^2^ = 0.315) (Figure 3). Set 1 was higher than Set 2 (*p* = 0.025) and Set 3 (*p* = 0.027). Set 2 was higher than Set 3 (*p* < 0.01). PS was greater compared to TS (*p* = 0.022). There was no significant difference between PS and SS (*p* = 0.106) or SS and TS (*p* = 0.522).

For the total training volume, there was a significant difference between interventions (*p* < 0.01, partial *η*^2^ = 0.42) (Table 2). PS was higher compared to TS (*p* < 0.01) and SS (*p* = 0.03). There was no significant difference between SS and TS (*p* > 0.99). For time efficiency, there was a significant difference between interventions (*p* < 0.01, partial *η*^2^ = 0.84). SS was more efficient compared to PS (*p* < 0.01) and TS (*p* < 0.01). PS was more efficient than TS (*p* < 0.01).

**Table 2:** Total training volume and time efficiency.

	Total training volume (Nm)	Time (min)	Time efficiency (Nm/min)
TS	8,426.4 ± 1,303.0*	8	1,053.3 ± 162.9*^.†^
SS	8,267.2 ± 1,323.8*	5	1,653.4 ± 264.8*
PS	9,691.1 ± 1,643.6	8	1,211.4 ± 205.5

Values were described as mean ± standard deviation. *p < 0.05 vs. PS. ^†^ < 0.05 vs. SS. PS, paired-set; SS, super set; TS, traditional set.

### 3.4 Subjective muscle fatigue, subjective general fatigue, DOMS, heart rate

For subjective muscle fatigue, there was no significant interaction (*p* = 0.23, partial *η*^2^ = 0.13) and no main effect for interventions (*p* = 0.24, partial *η*^2^ = 0.11), but there was a significant main effect for time (*p* < 0.01, partial *η*^2^ = 0.93). It increased after interventions [median values (interquartile range): TS; from 2 (2–3) to 8 (6–8), SS; from 2 (1–2) to 8 (6–9), PS; from 3 (2–3) to 8 (6–8), *p* < 0.01)].

For subjective general fatigue, there was no significant interaction (*p* = 0.59, partial *η*^2^ = 0.09) and no main effect for interventions (*p* = 0.59, partial *η*^2^ = 0.09), but there was a significant main effect for time (*p* < 0.01, partial *η*^2^ = 0.93). It increased after interventions [median values (interquartile range): TS; from 2 (1–3) to 6 (5–7), SS; from 1 (0–2) to 6 (5–8), PS; from 2 (1–2) to 7 (6–7), *p* < 0.01)].

For DOMS, there was no significant interaction (*p* = 0.33, partial *η*^2^ = 0.18), no main effect for interventions (*p* = 0.74, partial *η*^2^ = 0.05), and no main effect for time (*p* = 0.10, partial *η*^2^ = 0.21) (TS; 24 h 4.2 ± 1.7, 48 h 3.7 ± 2.6, SS; 24 h 4.5 ± 2.1, 48 h 3.2 ± 2.6; PS; 24 h 4.4 ± 2.0, 48 h 4.4 ± 1.8).

For heart rate, there was no significant interaction (*p* = 0.99, partial *η*^2^ < 0.01) and no main effect for intervention (*p* = 0.54, partial *η*^2^ = 0.11), but there was a significant main effect for time (*p* < 0.01, partial *η*^2^ = 0.97). It increased after interventions (TS; from 98.9 ± 12.9 bpm to 149.8 ± 12.2 bpm, SS; 100.8 ± 15.1 bpm to 151.2 ± 16.0 bpm, PS; from 96.7 ± 15.6 bpm to 148.0 ± 17.0 bpm, *p* < 0.01).

## 4 Discussion

The present study examined the effects of three different resistance training methods on muscle fatigue of the quadriceps and hamstrings. The muscle strength of the hamstrings significantly decreased in SS and PS, but not in TS. In addition, the muscle strength of the quadriceps decreased in all interventions without any significant difference between the interventions. TS is a successive resistance training method, and in this study, the hamstrings were trained, followed by quadriceps training for 3 sets. Therefore, in TS, the hamstrings had at least 270 s of intervals between the end of training and the measurement of muscle strength (three 60 s rest intervals and three 30 s quad resistance training). In contrast, for the other interventions and quadriceps, muscle strength measurements were performed within 90 s after the end of the resistance training. Previous studies reported that untrained men required 120 s for full recovery of muscle strength (18, 25). Therefore, it is possible that in the hamstrings of the TS, even if muscle fatigue occurred during resistance training, a decrement in muscle strength may be recovered by intervals between the end of training and the muscle strength measurement.

In all three interventions, training volume significantly decreased from the second set in the hamstrings and the third set in the quadriceps. Robbins et al. (20) examined training volume during resistance training with bench pull and bench press. They reported that the training volume of TS and SS decreased significantly from the second set. In addition, they reported no difference in total training volume between TS and SS, but SS had higher time efficacy (20). Paz et al. (7) reported that the training volume with bench press and seated row training of PS and TS significantly decreased from the second set and that the rate of decrease in the training volume was greater for TS than for PS. These results indicated that muscle fatigue may occur at the same timing regardless of resistance training method (TS, PS, or SS) in both previous studies of isotonic multi-joint training and the present study of isokinetic single-joint training.

In this study, the work-rest ratio per muscle was 1:2, 1:3, and 1:5 for TS, SS, and PS, respectively. Gentil et al. (26) randomized untrained men to one of two groups: a short-rest intervals group with a work rest ratio of approximately 1:3 and a long-rest intervals group with a work rest ratio of approximately 1:6. Both groups trained with 2 sets of 8–12 repetitions until volitional fatigue. They showed no significant difference in muscle strength after 12 weeks of resistance training between the groups. The results indicated that a 1:3 work-rest ratio may sufficiently recover in untrained men. However, the duration of the rest intervals in untrained men depends on the level of exertion (18, 27). When exercise is performed with maximal effort, a longer rest interval is required (18, 28). In the present study, participants performed maximal isokinetic muscle contraction with 3 sets of 10 repetitions, and the subjective muscle fatigue reached very high (NRS of 8 points). These results indicated that in the present study, in which all interventions were performed at maximal effort, the rest intervals in PS could be better than in SS and TS, and as a result, the total training volume was the highest in PS.

A lack of time is a common barrier to engagement in resistance training (29–31). Thus, it is important to improve time efficiency to increase adherence to resistance training. In this study, time efficiency was higher for SS, PS, and TS, in that order. Previous studies suggested SS for agonist and antagonist muscles is time-efficient compared to TS and PS (20, 32–34). Interestingly, the results of the present study showed no significant difference in subjective muscle fatigue and general fatigue, DOMS, or heart rate between interventions. These results support the utility of SS in individuals with limited training time. On the other hand, PS had the highest value for total training volume, which is an important factor for the long-term effects of resistance training. To our best knowledge, no studies have examined the effects of total training volume and time efficiency of SS and PS on long-term resistance training, and further studies should be conducted on the long-term effects of PS and SS.

This study had several limitations. This study was conducted on untrained, healthy men. The effectiveness of resistance training is influenced by gender and training history (18, 35). This study also compared the effects of immediate resistance training. The long-term effects of TS, SS, and PS on muscle strength and hypertrophy should be examined. In addition, this study targeted interventions to the flexor and extensor muscles of the knee joint. The influence of different resistance training methods on other muscles and multi-joint training needs to be examined.

In conclusion, this study showed that muscle strength of the hamstrings did not significantly decrease in TS, but did so in SS and PS. The muscle strength of the quadriceps significantly decreased in all interventions. Training volume significantly decreased with increasing sets in all interventions. Total training volume was the highest in PS, and time efficiency was the highest in SS. PS may be useful for individuals with sufficient time for resistance training because of higher training volume compared with other interventions, while SS may be useful for those with limited time due to better time efficiency.

## Data Availability

The raw data supporting the conclusions of this article will be made available by the authors, without undue reservation.
